# Review of Soft Robotic Gloves and Functional Electrical Stimulation Affecting Hand Function Rehabilitation for Stroke Patients

**DOI:** 10.3390/biomimetics11020104

**Published:** 2026-02-02

**Authors:** Xiaohui Wang, Yilin Fang, Zhaowei Zhang, Xingang Zhao, Dezhen Xiong, Junlin Li

**Affiliations:** 1 State Key Laboratory of Robotics and Intelligent Systems, Shenyang Institute of Automation, Chinese Academy of Sciences, Shenyang 110016, China; xiaohuiw@mail.ustc.edu.cn (X.W.); fangyilin@sia.cn (Y.F.); zhangzhaowei@sia.cn (Z.Z.); zhaoxingang@sia.cn (X.Z.); 2 University of Chinese Academy of Sciences, Beijing 100049, China

**Keywords:** soft robotic glove, functional electrical stimulation, hybrid hand rehabilitation system, stroke, hand rehabilitation

## Abstract

Stroke often results in impaired hand motor function, making effective hand rehabilitation essential for restoring activities of daily living (ADLs). Motor rehabilitation and neurorehabilitation are two major pathways to functional recovery. Rehabilitation gloves have proven to be effective tools for motor rehabilitation, and among them, soft robotic gloves (SRGs) have emerged as a research focus due to their lightweight design and inherent safety. Functional electrical stimulation (FES), which applies electrical currents to muscles and nerves, shows promise in promoting motor neural reorganization and restoring muscle strength in the hands of stroke survivors. The technologies applied to hand rehabilitation must possess the characteristics of safety, comfort, and practicality, while overcoming critical challenges such as portability, user-friendliness, and wearability. Motivated by the rehabilitation needs of post-stroke patients, this paper reviews recent advances in SRGs, FES, and hybrid hand rehabilitation systems (HHRSs) for hand rehabilitation, systematically examining progress in actuation strategies, intention sensing, and control algorithms across these three technologies. Furthermore, the limitations and technical challenges of current HHRSs are analyzed and four key future research directions are identified to pave the way for further development in this field.

## 1. Introduction

The Global Burden of Disease, Injuries, and Risk Factors Study (GBD) identifies stroke as a primary cause of mortality and disability globally [1]. About 75% of stroke survivors face motor impairments, with 55% to 75% showing ongoing dysfunction in the upper limb and hand [2,3]. Hand function constitutes roughly 90% of upper limb function [4,5]. Hand function is essential for performing activities of daily living (ADLs) [6,7,8,9]. The impairment affects patients’ quality of life, self-care capabilities, and mental health, while also elevating the burden on families and society [10]. Post-stroke hand rehabilitation is crucial for restoring independent living skills. Traditional rehabilitation relies on specialist therapists, requires prolonged treatment periods and substantial costs, and is often concentrated in specific medical institutions [11,12]. As the patient population grows, conventional manual rehabilitation faces challenges in meeting the increasing demand. Additionally, hand recovery relies on repetitive, task-specific training models. Based on the above requirements, the application of robotic technology and functional electrical stimulation (FES) in post-stroke hand rehabilitation has garnered considerable attention.

Currently, hand rehabilitation robots are mainly categorized into rigid exoskeletons and soft exoskeletons [13]. Rigid exoskeletons represent the initial devices utilized in rehabilitation. However, they are generally bulky, averaging around 500 g in weight [14], and exhibit limited adaptability to varying hand sizes. These limitations may result in joint misalignment, decreased wearing comfort, and reduced bionic properties [15,16], which complicates the simultaneous achievement of multi-degree-of-freedom (multi-DOF) actuation and portability. Soft exoskeletons employ flexible, deformable materials to improve bionic properties and compliance, thereby minimizing joint misalignment and related injuries [17]. Their lightweight, safe, and portable features facilitate home-based rehabilitation, consequently reducing treatment costs [18]. Studies show that soft exoskeleton robots effectively enhance hand function in stroke patients [19,20,21,22], supporting home-based use, extending training duration, and aiding in the recovery of finger flexion, extension, and grasping abilities, while also allowing for personalized training and monitoring of rehabilitation data. Soft exoskeletons demonstrate advantages in human–machine interaction, multi-degree-of-freedom control, and power-to-weight ratio, making them more suitable for hand rehabilitation applications compared to rigid solutions [23,24]. In this study, the soft exoskeletons investigated are primarily soft robotic gloves (SRGs). For severely paralyzed patients, SRGs mainly offer passive assistance and face challenges in promoting active muscle engagement, highlighting a notable limitation in current technology.

In addition, the integration of FES into traditional rehabilitation enhances hand recovery in stroke patients [25]. FES is a technology utilized in the development of neural prostheses aimed at facilitating hand rehabilitation following a stroke [26]. The application of electrical currents to damaged nerves or muscles mimics normal neural conduction, eliciting muscle contractions that help prevent atrophy and promote motor neural remodeling [27]. FES employs either implantable or surface electrodes in conjunction with functional tasks to stimulate motor neurons using low-level pulses, thereby enhancing upper limb motor functions, including grasping and reaching [28,29]. The direct benefits encompass decreased muscle spasticity, increased joint range of motion, and postponed disuse atrophy [30,31]. However, FES depends on intact motor nerves, and repetitive stimulation may induce muscle fatigue, and muscles demonstrate highly nonlinear responses to stimulation, which diminishes movement accuracy and repeatability [32,33,34].

SRGs are effective for hand rehabilitation training, allowing for repetitive motions and fine manipulations with restorative functions. FES enhances the reorganization of the hand’s motor system and stimulates muscle activity more effectively. SRGs can overcome the limitations of FES by integrating both methodologies to leverage their advantages while mitigating their disadvantages. This hybrid hand rehabilitation system (HHRS) combines electrical stimulation to induce muscle perception with soft mechanisms to assist in motor tasks such as grasping, thereby improving rehabilitation efficacy. The system mitigates interference through closed-loop control, thereby enhancing control accuracy and reducing muscle fatigue. This hybrid strategy may concurrently enhance joint range of motion and improve hand closure and opening abilities, thereby increasing patients’ functional independence [35]. Research in this area is currently constrained. Cardoso et al. investigated the contributions of soft robotics and FES, as well as their integration, in the rehabilitation of hand function among individuals with SCI [26]. The authors concluded that soft robotics and FES wearable devices are promising technologies for improving hand function recovery in patients with spinal cord injuries. Nonetheless, challenges remain in user intent detection, portability, calibration, and the reliable evaluation of functional outcomes. Patel and Bachkaniwala demonstrated that a hybrid rehabilitation system integrating FES and SRGs was more effective than monotherapy in enhancing various hand function tasks in stroke patients [35]. Meanwhile, this HHRS is still at its early stage of research development [36]. Table 1 presents a summary of pertinent research reviews conducted over the last eight years.

**Figure 1:**
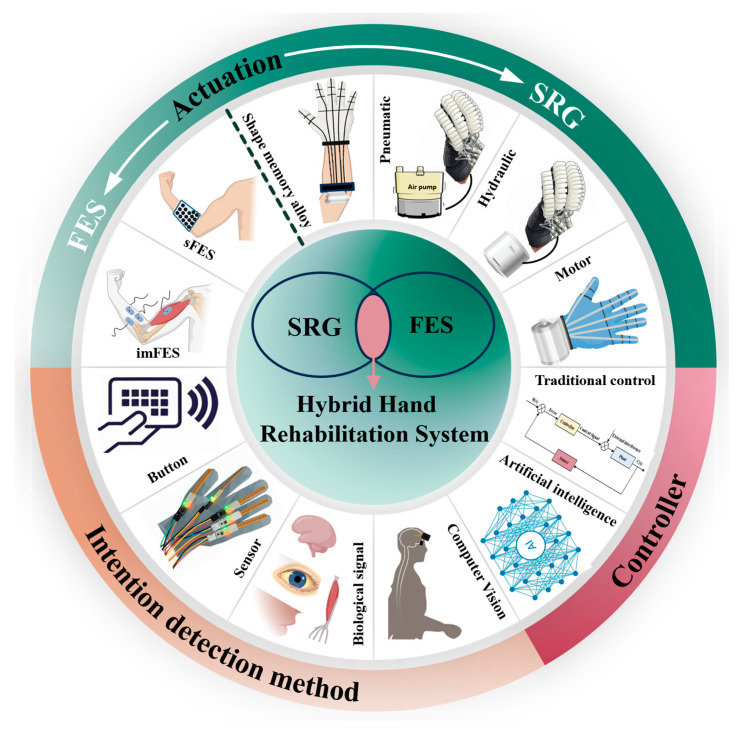
Main contents of the review. Research on actuation, patient intention detection, and control algorithms for SRGs, FES, and HHRS.

Although there are systematic reviews on SRGs for post-stroke hand rehabilitation and separate reviews on FES-based rehabilitation, along with some work summarizing the combined use of FES with robotic gloves, there is currently no comprehensive review that simultaneously compares SRGs, FES, and hybrid rehabilitation approaches for post-stroke hand rehabilitation. This paper presents a systematic review of three major hand rehabilitation technologies for post-stroke patients, including SRGs, FES, and HHRSs. Recent research progress is summarized from the perspectives of actuation strategies, intention detection, and control algorithms (Figure 1). Furthermore, key technical and application challenges in post-stroke hand rehabilitation are analyzed, such as the development of portable, intelligent, and wearable systems for home-based rehabilitation, and four important future research directions are proposed.

## 2. Actuation Type

Hand rehabilitation devices aim to aid patients with strokes or neurological injuries in recovering fine motor skills, including grasping, pinching, and stretching. The actuation module in the hand rehabilitation system is a key component that dictates the compliance, safety, and wearability of the rehabilitation apparatus. The actuation method is a crucial factor affecting system structural design and control strategies, directly influencing their application contexts and portability.

### 2.1. Actuation Types of SRGs

Recently, advancements in SRGs employing flexible materials and soft actuation technology have made substantial progress. Now, SRGs predominantly utilize four forms of actuation: electric motor, hydraulic, pneumatic, and shape memory alloy (SMA). Table 2 delineates the advantages and disadvantages of these four separate actuation mechanisms of SRGs.

#### 2.1.1. Motor Actuation

Motor-driven systems are the most common actuation method for rehabilitation robots at present [42]. Motors allow for the control and modulation of movement in flexible structures by converting electrical energy into mechanical energy. They offer advantages such as accurate control, a compact design, and low noise levels [43]. Based on their sources of actuation, they predominantly comprise tendon-driven and the three-layer sliding spring (TLSS)-based mechanisms. Among these, tendon-driven mechanisms mainly include cable-driven and string-driven approaches.

Tendon drives typically employ low-creep inelastic cords to simulate hand tendons, offering advantages in remote and compliant actuation while achieving high precision in force regulation [44,45]. Connecting micro motors to fingertips through tendons allows motor rotation to facilitate finger movement, resulting in lightweight, compact constructions with compliant control [46,47]. This work examines force transmission and mechanism optimization, namely by minimizing friction through enhanced tendon and pulley configurations [48]. The topology typically relies on N or N + 1 configurations, where N represents the DOF. In N + 1 systems, each joint incorporates two tendons to enable flexion and extension [49,50]. The SRG based on this mechanism can provide multi-degree-of-freedom movement and promote finger dexterity [44,51,52].

**Figure 2:**
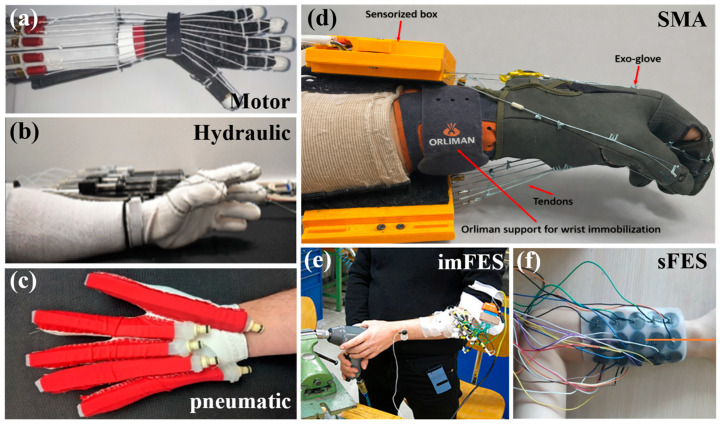
The actuation types of SRGs and FES. (**a**) A motor-TSA-driven SRG [53]. Reproduced with permission from Li et al., Biomimetics, Published by MDPI, 2023. (**b**) HFAM-based wearable glove [77]. Reproduced with permission from T. N. Do et al., IEEE Access, Published by IEEE, 2020. (**c**) A pneumatic SRG [87]. Reproduced with permission from McCall et al., Journal of NeuroEngineering and Rehabilitation, Published by Springer Nature, 2020. (**d**) A SMA-driven SRG [115]. Reproduced with permission from Copaci et al., IEEE Access, Published by IEEE, 2024. (**e**) Hand rehabilitation equipment with implant FES [138]. Reproduced with permission from Hahne et al., Frontiers in Neurorobotics, Published by Frontiers Media S.A., 2016. (**f**) Hand rehabilitation equipment with surface FES [150]. Reproduced with permission, Copyright 2021, Taylor & Francie.

The twisted string actuator (TSA) transforms motor rotation into linear motion through the twisting of strings, not only augmenting output force but also streamlining SRGs (Figure 2a) [53,54,55,56]. TSAs facilitate high-precision, repetitive linear motion, making them suitable for assistive SRGs that require meticulous finger control. However, it has limited load-bearing capacity and is not suitable for high output force. Nylon cords serve as artificial tendons, with motors exerting force on these cords to actuate fabric SRGs, facilitating the movement of the fingers for grasping aid [57,58]. Devices such as the “Bio-exoskeleton Glove” facilitate accurate and powerful griping through tension lines, thereby enhancing patients’ ADLs [59].

Cable-driven systems enable finger flexion and extension by tensioning cables, commonly Bowden cables [26]. Bowden cables allow for intricate routing paths that enable actuator to be located remotely from the end-effector, thereby lessening the robot’s bulk and weight [47,60,61,62]. However, its compliance and comfort are inferior to those of the rope-driven system. Bowden cables are used as tendons and structural elements in the “Mano” hand exoskeleton, which reduces weight and exposes the palm and fingertips for improved touch and comfort [63]. Another system integrates twisted-cable actuation with Bowden cables for finger flexion [64]. “HEXOES” utilizes a flexible cable-driven mechanism to autonomously actuate and perceive all 10 DOFs of the hand [65].

TLSS utilizes layered spring blades linked by rigid elements, facilitating a hand-contouring framework characterized by significant compliance and adaptability [66]. However, it has the disadvantages of nonlinearity and limited adjustability. Because the bending springs store energy, they are particularly suitable for individuals with early-stage poststroke spasticity, which helps with finger extension. The natural flexion and extension of three joints are compactly provided by the compliant springs of TLSS with a single DOF [67]. The improved “Tenoexo” exoskeleton utilizes a ball screw mechanism to actuate sliding blades and integrates an electric thumb abduction/adduction module, supporting over 80% of daily activities [68].

#### 2.1.2. Hydraulic Actuation

Hydraulic actuation facilitates the deformation of soft structures by adjusting fluid pressure, offering greater output torque than cable or pneumatic systems, making it more suitable for high-power applications [69,70,71]. However, the fluid medium increases weight and occupies volume [72,73], constraining its application in hand rehabilitation. Therefore, a miniature hydraulic drive system was developed for incorporation into a wearable haptic SRG [74]. Existing studies have developed miniature hydraulic modules integrated into wearable haptic gloves, which utilize artificial muscles to achieve finger bending and employ micro-pressure sensors for closed-loop control. Notably, this system functions autonomously, eliminating the need for external wiring [74]. Hydraulic soft actuators, consisting of thin-walled elastic bladders and fiber-reinforced polymers, can perform precise bending, twisting, and stretching movements, facilitating home-based rehabilitation [23,75,76]. A soft hydraulic filament artificial muscle (HFAM) was developed with high stretchability, fast response, and scalability, thereby improving grasping performance (Figure 2b) [77]. Nonetheless, liquid leakage presents safety risks, serving as a principal rationale for the restricted quantity of such devices [23,69,78].

Liquids provide superior positional control as a medium compared to compressible gases. When utilizing various liquid-driven systems, it is worth considering the influence of aspects such as the operational environment and temperature of the SRG on liquids, along with the liquid’s effects on the surroundings, based on the intrinsic qualities of the selected fluid.

#### 2.1.3. Pneumatic Actuation

Despite the maturity of motor-driven technologies, pneumatic-driven SRGs have attracted heightened interest owing to their absence of overload risks [17,79,80]. Pneumatic SRGs are lightweight, adaptable, and simple to install, rendering them appropriate for hand assistance and rehabilitation. They regulate SRG motion by controlling gas flow and pressure, employing airbags or pliable structures to facilitate deformation and movement. Pneumatic actuators provide benefits in force output, control accuracy, and weight-to-torque ratio, rendering them the optimal selection for hand rehabilitation robots [81]. Pneumatic soft actuators can be principally classified into four categories based on their structural design: (i) Pneumatic Artificial Muscles (PAMs), such as McKibben muscles; (ii) Fluid-elastic actuators (FEAs), often referred to as soft elastic actuators (SEAs), including PneuNet actuators or soft bending actuators (SBAs); (iii) Fabric actuators; (iv) 3D-printed actuators [82].

Pneumatic SRGs utilizing McKibben or analogous pneumatic muscle-like actuators facilitate rehabilitative movements of the hands [38,83,84]. The McKibben actuator consists of a flexible elastic tube enveloped in woven yarn or fibers, generally exhibiting linear motion, with force and displacement modifiable through air pressure. A Japanese laboratory, engaged in extensive research on McKibben muscles, has developed a thin-profile McKibben pneumatic actuator to mitigate the issue of restricted finger mobility and difficulty wearing the device in patients with spasticity [38]. Koizumi et al. [79] developed a SRG that facilitates finger extension and flexion with their concept “three-point bending structure” and “flat woven muscle” principles. This design preserves compactness at the wrist and hand, effectively addressing the constraints of size, weight, and extension force characteristic of conventional rehabilitation gloves.

Soft elastomer-based actuators consist of interconnected chambers that are generally constructed from pliable materials such as silicone or elastomers and allow air to flow freely between them (Figure 2c) [85,86,87]. The interrelated design of PneuNet structures facilitates intricate synchronized motions across many chambers [88,89], whereas bellows-shaped structures permit longitudinal expansion under overpressure [90]. PneuNet-based SEAs incorporated into neoprene gloves effectively, affordably, and conformably emulate finger movements [91]. Research demonstrates that the 20 Shore A of PneuNet version, among PneuNet actuators fabricated with varying silicone stiffness and extended McKibben actuators, exhibits superior performance in SRGs designed [92]. The PneuNet SRG facilitates more natural and precise finger movements while reducing operational pressure through the addition of chambers and external threaded reinforcement components [93]. Sandoval-Castro et al. [94] incorporates non-stretchable layers filled with chia and quinoa particles to regulate actuator stiffness, providing innovative perspectives for hand rehabilitation applications. Moreover, fiber-reinforced silicone may be utilized to manufacture SRGs [95].

Fabric-based pneumatic actuation has been implemented in hand exoskeletons, demonstrating biocompatibility, flexibility, and durability, with proven efficacy in ADLs for chronic stroke patients [96,97,98,99,100]. The use of flexible actuators with textile materials facilitates low-pressure grip support for stroke patients while preserving the range of motion in finger joints [72]. A pneumatic fabric glove that emulates honeycomb structures diminishes reliance on air chamber deformation while maintaining functionality at low pressures [21]. The fabric-based ExHand-exoskeleton aids stroke patients in grasping things necessary for ADLs [101]. Suulker et al. established that fabric-based SRGs produce greater output forces compared to elastomer SRGs, including silicone [102].

Soft pneumatic actuators based 3D printing technology facilitate rapid prototyping iterations and can be assembled in a bottom-up manner by printing flexible and elastic materials. As a result, they are capable of constructing SRGs with intricate designs [103]. In stroke therapy and daily activity assistance, 3D-printed soft-elastic composite actuators (SECA) and ring-enhanced soft actuators promote finger flexion and extension while accommodating various hand sizes, allowing for personalized designs [104,105]. Advancements in printing technology have enabled the fabrication of pneumatic SRGs with silicone-platinum materials, which provide lightweight, comfortable, safe, and user-friendly solutions [106]. 3D-printed pneumatic soft finger actuators enable iterative testing, lower expenses, and promote the development of wearable medical equipment, improving rehabilitation results and quality of life for stroke patients [107].

Single-material systems encounter constraints including inadequate stress tolerance, limited structural complexity, and elevated input pressure. In contrast, integrating several materials mitigates certain limitations while maintaining their exceptional characteristics. For example, integrating fabric skin designs with silicone reservoirs and anisotropic elastic outer layers facilitates enable reconfigurable flexible structures [108]. Moreover, hydraulic and pneumatic systems necessitate stringent maintenance to avert leakage and corrosion.

#### 2.1.4. SMA Actuation

SMA represent a distinct category of smart materials that can deform at specific temperatures and possess the ability to return to their original configuration upon heating. In SRGs, SMA is commonly utilized to facilitate component deformation or motion, capitalizing on its shape memory effect and super elasticity [109,110]. This facilitates lightweight, energy-efficient designs that enhance human–machine interaction performance [111,112].

Researchers at Tehran University in Iran have developed a wearable SRG utilizing SMA filaments. Guide rails affixed at finger joints dictate the contraction trajectory of the SMA, with forces produced by the SMA actuator offsetting inadequate finger muscle strength to facilitate flexion and extension movements within a specified range [113]. To improve adaptability and safety in environmental interactions, Serrano et al. proposed an modified SMA-driven SRG that enables relative motion of the thumb to allow patients to execute more intricate rehabilitation or ADLs gestures, thereby enhancing their independence in daily tasks [114]. SMA-driven rehabilitation SRGs demonstrate slow, smooth and controllable motion upon activation, while ensuring full control transparency when inactive to improve patient comfort and acceptance (Figure 2d) [115].

Pneumatic artificial muscles and SMA-driven systems are the most commonly used novel actuation methods in hand rehabilitation robots, exhibiting excellent comfort and safety profiles. SMA-driven SRGs, while demonstrating inferior grasping performance relative to pneumatic drives and facing challenges such as motion lag and high nonlinearity [116], possess notable advantages, including biocompatibility, high energy density, and substantial output force, indicating their potential as intelligent actuators [117]. In the coming decade, research in SRGs will persist in investigating new soft actuation techniques and their creative applications [118].

### 2.2. Actuation Types of FES

Stroke constitutes a significant neurological disorder [119]. Nerve injury in the hand results in inadequate innervation, impairing the muscles’ ability to produce force effectively. Therefore, both neural and muscular rehabilitation are of equal significance. The fundamental principle of neural rehabilitation is neuroplasticity [120,121]. FES produces muscle force via pulsed stimulation, promoting patient independence while effectively strengthening muscles and enhancing sensory feedback. It inhibits arm muscle atrophy and remodels the hand’s motor nervous system, thereby supporting neuromuscular re-education and motor relearning following a stroke [41,120,122,123,124]. The intensity of FES can be adjusted flexibly. Two modalities are available for finger flexion and grasping: subcutaneous electrodes and surface electrodes.

#### 2.2.1. Subcutaneous FES

Subcutaneous FES refers to a form of functional electrical stimulation in which electrodes are implanted beneath the skin while remaining extramuscular and extraneural, delivering controlled electrical pulses to activate target neuromuscular structures for the restoration or assistance of functional movements. Such systems can be implemented as either percutaneous or fully implantable configurations.

Percutaneous FES entails the insertion of filament electrodes into muscle tissue adjacent to the target nerve through subcutaneous injection. The electrodes are positioned temporarily and are generally employed for brief interventions [125,126]. Although surgery is not necessary, the risk of infection persists [127]. The Cleveland FES Center has utilized percutaneous FES safely for extended periods, ranging from months to years, in certain stroke patients. Chae et al. conducted evaluations demonstrating that percutaneous hand FES enhances hand motor function in stroke patients [128,129]. An experiment involving the implantation of electrodes in the forearm muscles of four chronic stroke patients for grasping and releasing tasks demonstrated that this system can repeatably perform functional activities, enhance motor relearning, and function as a transitional technology for implantable neural prostheses [128].

The electrode of implantable FES (imFES) are surgically implanted into muscles to facilitate targeted activation. Electrode arrays are constructed by assembling metal microfilaments or semiconductor probes. The current necessary to elicit muscle contraction does not surpass 25 mA [32]. The FES system exhibits high precision, low stimulation intensity, and accurate configuration capabilities [130], facilitating repeated muscle activation and control of deep muscles. However, surgical risks encompass wound infection and electrode fracture, while the positioning of electrodes is not adjustable. This makes the approach more appropriate for longitudinal studies and long-term applications [28,131].

Implantable microstimulators [132,133,134] and multi-channel implantable pulse generators [135,136,137] are appropriate for stroke and SCI patients who have been meticulously evaluated for excessive flexor spasticity, primarily aimed at restoring autonomous function rather than providing daily assistance. Titanium transcutaneous implantable electrodes, as proposed by Hahne et al. [138], mitigate skin complications and electrode displacement by establishing a direct connection to external amplifiers, eliminating the need for wireless transmission (Figure 2e). ImFES selectively activates deeper muscle groups, facilitates control over multiple joints, and produces various movement patterns. High-frequency stimulation through nerve sleeve electrodes reduces spasticity and enhances motor function in the hand and arm of stroke patients [139].

Subcutaneous FES directly stimulates nerves, enabling a single electrode to effectively target several hard-to-reach muscles. The advantages include high stimulation selectivity, ease of operation, and broad applicability; however, the presence of an incision poses a risk of infection. Subcutaneous FES facilitates selective control of specific deep muscles in patients needing long-term rehabilitation or those with severe strokes, thereby improving rehabilitation training outcomes and assistive functions.

#### 2.2.2. Surface FES

Surface FES (sFES) is an established non-invasive method employed for motor rehabilitation in patients with paralysis [140,141,142]. By delivering electrical pulses to the skin surface over targeted nerves or muscles, sFES stimulates paralyzed muscles to regain motor function in stroke patients. In typical sFES systems, these electrical pulses are generated by an external programmable stimulator and delivered as controlled current pulses through surface electrodes placed on the skin overlying the target neuromuscular structures. Utilizing a superficial electrode array directly applied to the forearm muscles and nerves improves selectivity and accuracy in finger movements, overcoming the limitations of traditional surface electrodes characterized by poor selectivity and limited control precision. sFES can be applied in clinical or home environments [142], generally involving currents (2–120 mA) that exceed those of implanted electrodes but remain safe for the majority of stroke patients. Early post-stroke intervention (within 2 months) enhances patients’ ADLs.

Early sFES [143,144,145] encountered limitations such as inadequate selectivity, system complexity, tingling sensations, muscle fatigue, and suboptimal hardware and software. Nevertheless, multi-channel sFES has shown promise in enhancing upper limb and hand function following a stroke [29,146,147,148]. MyndMove^TM^ incorporates multi-channel FES technology and has demonstrated feasibility within an ambulatory setting, providing an option for the rehabilitation of severe chronic stroke upper limb impairments [148]. Gritsenko et al. integrated surface electrodes with a modified Impact Cuff stimulator to facilitate activation of wrist and finger extensor muscles through sFES, allowing patients to choose between grasping or opening using buttons, thus enhancing upper limb and hand functionality [149]. A hand FES system utilizing a high-density electrode array (comprising 32 electrodes with motion feedback sensors) has shown potential for finger movement control during testing on healthy subjects [140]. Wang et al. utilized a 5 × 5 array of multi-channel surface FES to selectively stimulate fingertips, thereby enabling precise motor control for activities of daily living (Figure 2f) [150]. Kapadia et al. utilized a four-channel programmable sFES system for reaching and grasping rehabilitation in 50 stroke patients, which can implement the personalized stimulation protocols within 10–15 min, providing safe and practical therapy [39].

Contralateral Controlled Functional Electrical Stimulation (CCFES) has been clinically shown to enhance hand movement control and dexterity in stroke patients by facilitating simultaneous bilateral hand movements [151,152], which is accomplished through the engagement of the non-paralyzed upper limb to administer neuromuscular electrical stimulation to the paralyzed upper limb. Utilizing an 8-channel electrode array, each gesture is assigned to a specific FES channel, and three stimulation channels utilizing bipolar waveforms are used to reduce muscle fatigue and tingling sensations [152]. CCFES constitutes a more advanced form of electrical stimulation in comparison to traditional neuromuscular electrical stimulation [153]. Fu et al. [154] introduced a home-based therapeutic approach that combines CCFES with video games for hand rehabilitation. Initial clinical trials revealed enhancements in both motor abilities and cognitive function among participants.

In recent years, research on FES has progressively transitioned toward sFES, with the majority of commercially available hand rehabilitation devices employing it. Although its stimulation accuracy is inferior to that of implantable electrodes, sFES provides benefits such as non-invasiveness, straightforward replacement, rapid deployment, cost-effectiveness, and suitability for home use and maintenance [155]. Early implementation of sFES in stroke patients can harness central and peripheral neural plasticity to facilitate functional recovery of the hand and upper limb. Popovic et al. proposed that subcutaneous FES is not advised for patients who are capable of effectively utilizing sFES daily [32].

### 2.3. Actuation Types of HHRSs

SRGs and FES are both effective and complementary methods for stroke-induced hand rehabilitation, capable of reducing muscle fatigue and spasticity, thereby enhancing recovery outcomes for patients [156,157]. HHRSs that combine both technologies have been developed [157]. However, research in this area is still limited.

The Intelligent Haptic Robotic Glove (IHRG) rehabilitation system consists of a wire-driven SRG and sFES from the Motionstim8 neurostimulator. The system detects patient intent using glove sensors, stimulates muscle contraction and activates SRG through FES, and engages the SRG to facilitate finger movements in stroke patients [158]. The IHRG rehabilitation system maintains a balance of control between FES and tendon-driven SRG, employing dual-channel FES to regulate glove contractions through activation or deactivation, which can preserve hand grasping and minimize muscle fatigue [159]. The HEXaFES system utilizes sFES with SRGs to assess muscle fatigue, thereby postponing its onset and improving rehabilitation results, but its application is presently restricted to hemiplegic patients [160]. The TipStim glove, a wearable device utilizing composite elastic textile materials, incorporates fingertip electrodes and bidirectional pulsed current to stimulate both superficial and deep muscles, thereby improving hand sensation and function in individuals with upper limb paralysis [161]. Nam et al. developed an electromyography-signal-driven exoskeletal neuromuscular system that incorporates neuromuscular electrical stimulation (NMES), pneumatic muscles, and exoskeleton technology to mitigate finger and upper limb spasticity and to restore voluntary motor function [162]. This system utilizes micro air compressors to actuate pneumatic soft actuator hand module, facilitating finger flexion and coordinated movements while reducing mechanical scale and power demands to attain near-normal muscle coordination. Hybrid systems use FES to enhance voluntary muscle recruitment while compensating for its limited torque through assistance from SRG, resulting in improved finger flexion compared with FES-only systems [163].

SRGs and FES have shown effectiveness in aiding and rehabilitating hand function. HHRSs integrate the benefits of both systems, providing wearability, safety, and tremor and spasticity suppression, as well as the capacity to remodel motor neurons. They improve precision and independence of finger movements in stroke patients, enhance rehabilitation outcomes, and facilitate more effective rehabilitation.

Figure 2 shows the actuation types of SRGs and FES.

## 3. Patient Intention Detection

The interactive control between hand rehabilitation robots and patients is a pivotal research focus in this domain. SRGs need to engage with limbs that exhibit motor function impairments, whereas patients necessitate autonomous movement perception. Interactive control offers patients a secure, comfortable, natural, and dynamic training environment, mitigating counterforces induced by atypical muscular activity such as spasticity or tremors, thus safeguarding limbs from further harm. Concurrently, interactive control employs sensors to identify patients’ movement intentions, promoting active engagement in training and improving rehabilitation results. Human intent detection underpins robots’ ability to deliver support customized to patient requirements [164]. Hand rehabilitation devices can facilitate finger movements, enhance neuronal remodeling, and expedite healing by anticipating movement intentions.

### 3.1. Patient Intent Detection and Control of SRGs

The methods for detecting human intent in SRGs are classified into four categories: button, sensor signals, biological signals, and computer vision.

#### 3.1.1. Button

Buttons [63,76,98,99,112,165,166] represent the commonly used trigger-based intent detection methods. The system provides straightforward, secure, and dependable control of hand movements. However, they lack reliance on authentic movement intent and therefore do not represent a real patient intent detection method.

Polygerinos et al. [76] developed a hydraulic SRG that utilizes mechanical switches on a control box to manually operate individual finger actuators. Yap et al. [99] utilized buttons to control a fabric pneumatic SRG that performs power grip, pinch grip, and tripod pinch grip motions, aiding stroke patients in ADLs.

**Figure 3:**
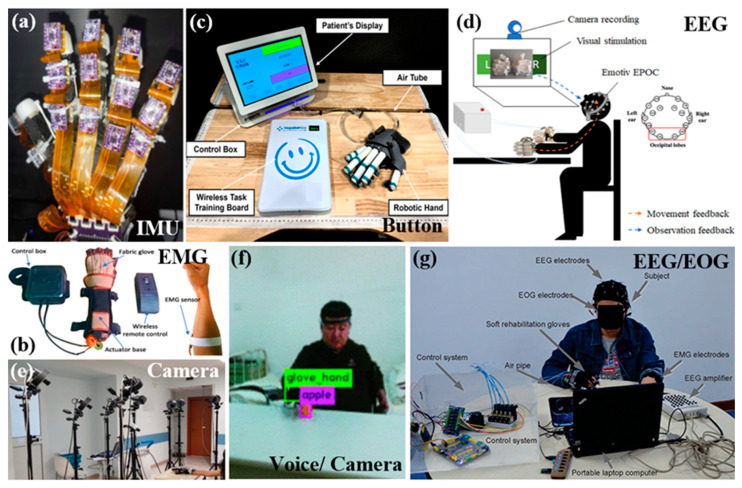
Patient intention detection methods of SRGs. (**a**) IMU sensors-based intention detection and control of SRG [53]. Reproduced with permission from Li et al., Biomimetics, Published by MDPI, 2023. (**b**) EMG-based intention detection of SRG [57]. Reproduced with permission from Ismail et al., HardwareX, Published by Elsevier, 2024. (**c**) Button-based intention detection and control of the SRG [105]. Reproduced with permission from Shi et al., Wearable Technologies, Published by Cambridge University Press, 2025. (**d**) SSVEP-based BCI of SRG [121]. Reproduced with permission from Guo et al., IEEE Transactions on Neural Systems and Rehabilitation Engineering, Published by IEEE, 2022. (**e**) Camera-based intention detection of SRG [199]. Reproduced with permission from Cordella et al., Journal of Biological Regulators and Homeostatic Agents, Published by Biolife SAS, 2020. (**f**) Voice/Camera-based intention detection of SRG [200]. Reproduced with permission from Chen et al., the 2020 39th Chinese Control Conference (CCC), Published by IEEE, 2020. (**g**) EEG/EOG-based intention detection of SRG [202]. Reproduced with permission from Zhang et al., Frontiers in Neurorobotics, Published by Frontiers Media S.A., 2016.

Intelligent button systems are being increasingly utilized for recognizing hand movement intent and controlling SRGs. Randazzo et al. [63] utilized a smartphone interface to control the “mano” hand exoskeleton, achieving ADL and rehabilitation, thereby enhancing hand neural plasticity and supporting home training. Santos et al. [165] developed the Nuada glove, which transitions between states via light taps on a smartwatch for gesture control. SRGs, when used in conjunction with wireless task boards, assist the grasping and extension of spastic fingers in stroke patients, and it has been shown to enhance grasping movements in ADLs and facilitate recovery of hand function (Figure 3c) [105].

#### 3.1.2. Sensors

The use of multiple sensors, such as force sensors, tactile sensors, flexible bend sensors, and inertial measurement units (IMU), facilitates the detection and control of patients’ hand movement intentions through the capture of hand movement information. Sensor-based intention detection facilitates precise monitoring of hand movement states and evaluation of rehabilitation progress, with pressure sensors being the most commonly utilized.

Pressure sensors [95,164,167,168] provide benefits including high accuracy, rapid response, reliability, small size, light weight, and straightforward installation. They facilitate accurate measurement and regulation of force while effectively mirroring the patient’s movement intention and condition. Pressure-based systems typically integrate thin-film or flexible force sensors into the glove’s palmar or fingertip regions. Typical signals collected by pressure sensors include contact force/pressure, force distribution, and grip force curves, which can be used for motion intention recognition and closed-loop force control. Hosseini et al. [167] developed the ExoTen-Glove, which integrates two TSAs with force sensors to deliver haptic feedback during virtual object grasping, thereby improving hand rehabilitation efficiency. Pressure sensors are utilized in silicone SRGs to assess finger positions and provide feedback [95]. Islam et al. [164] introduced a new approach employing force sensitive resistance sensors for the detection of hand movement intentions. Kottink et al. [169] used pressure sensors to detect the contact force between fingertip and objects and trigger flexion movements, experimentally demonstrating that the SRG can be used at home and increases patients’ training duration.

Human tactile perception facilitates object recognition and manipulation through the conversion of skin deformation into electrical impulses [170]. The lack of tactile feedback significantly affects the ADL and engagement of stroke patients. Thus, tactile sensors are extensively studied and utilized to restore hand sensation and enhance neuroplasticity. Tactile-sensing gloves often embed capacitive, piezoresistive, or textile-based tactile arrays on contact surfaces. Tactile sensors typically collect signals such as local pressure distribution, temperature, contact location, and texture information, which can be used to infer object properties and contact stability. Kim et al. [171] developed a textile glove incorporating palm-side sensing elements to deliver feedback regarding the hardness, humidity, and temperature of objects that are touched or grasped. Glauser et al. [172] introduced a SRG utilizing capacitive sensors for the capture of interactive hand postures. Ozlem et al. [173] employed capacitive sensors to record physician hand movements, subsequently controlling pneumatic rehabilitation gloves used by stroke patients through the Internet of Things, akin to mirror therapy rehabilitation. Song et al. [174] introduced a SRG that utilizes fingertip haptic feedback produced by electrostatic forces, which generate internal pressure and remove the necessity for an external air source.

Flexible bending sensors [23,95,111,112,175] convert bending angles into electrical signals, characterized by lightweight, stretchable, and highly sensitive properties, enabling real-time hand posture reconstruction. Flexible bending sensor–equipped gloves usually incorporate stretchable strain or bend sensors along finger dorsal or lateral surfaces. They typically provide bending angle, angle velocity, and posture sequence data, which can be used for gesture recognition and motion tracking. Polygerinos et al. [23] integrated electromagnetic (EM) tracking sensors into stretchable silicone strips affixed to each finger of a SRG to quantify finger flexion and extension. Xie et al. [111] incorporated a bending sensor layer into exoskeletons made of SMA to measure the bending angle of composite structures. Fiska et al. [175] utilized ten flexible bending sensors in conjunction with four thin-film piezoelectric sensors to assess post-stroke hand kinematics. Multi-sensor systems are extensively employed in studies to meet various hand rehabilitation requirements for stroke patients [108,127,176].

IMU provides benefits including rapid response, compact dimensions, and ease of wearability. In hand rehabilitation, IMU are frequently integrated into SRGs to reconstruct hand movements, monitor posture and motion, and implement gesture recognition. IMU-integrated gloves typically position miniature inertial modules on the dorsal side of the hand or along individual fingers to capture segmental motion information. The signals acquired from IMUs include tri-axial acceleration, angular velocity, and magnetic field intensity, which can be fused using sensor fusion algorithms to estimate hand orientation and motion trajectories. The GESTO glove, created by Baldi et al. [177], integrates inertial and magnetic sensors for hand motion tracking and capture. Li et al. [53,178] utilized 15 miniature IMU integrated into a TSA-driven SRG to predict finger joint angles and track whole-hand movements (Figure 3a). Additionally, IMU are often integrated with other sensors to acquire more precise and comprehensive data for hand rehabilitation.

Although sensors can capture comprehensive hand movement information, their design and integration pose several challenges. For pressure sensors, maintaining stable contact and avoiding signal drift due to skin deformation and sweat are critical issues. Tactile sensors require high spatial resolution and robustness to repeated friction, while ensuring user comfort. Flexible bending sensors must sensitivity and durability, as repeated bending may cause material fatigue and calibration drift. IMUs suffer from accumulated integration error and magnetic interference, requiring sensor fusion and drift compensation. Furthermore, multi-sensor systems increase wiring complexity and power consumption, and require effective data fusion algorithms to avoid redundant or conflicting signals. The performance of these sensors is affected by several factors, including skin–electrode contact quality, sensor placement consistency, mechanical deformation of the glove, temperature and humidity, and long-term wear-induced drift.

#### 3.1.3. Biological Signals

Recent advancements in intention recognition and control for SRGs have increasingly focused on human biological signals. This method facilitates enhanced prediction of motor intentions in stroke patients and promotes neural plasticity [179]. Neural synergies exhibit greater dimensionality and improved robustness relative to conventional muscle synergies [180]. Biological signals frequently utilized in research encompass the electroencephalogram (EEG), electromyogram (EMG), electrooculogram (EOG), and voice signals.

EEG-based intention detection utilizes brain–computer interfaces (BCI) to decode cortical activity directly, independent of muscular involvement, signifying an advanced area of research. Despite challenges including non-stationarity, nonlinearity, low signal-to-noise ratio, and susceptibility to interference, EEG has been shown to facilitate continuous intention decoding and control in SRGs, thereby enhancing hand movement in stroke patients [63,181,182,183,184]. Motor imagery-based brain–computer interfaces (MI-BCI) are capable of decoding sensorimotor rhythms (SMR) in the absence of physical movement, thus enabling the identification of hand movement intentions [176]. Visually induced MI is commonly utilized, with decoded signals transmitted to SRGs to facilitate intention-driven assisted hand movements [185,186,187]. A steady-state visually evoked potentials (SSVEP)-based BCI was used for intention detection to control a SRG for post-stroke hand rehabilitation, achieving outcomes comparable to those of an MI-BCI (Figure 3d) [121]. Non-invasive BCI provides benefits including safety, cost-effectiveness, and real-time functionality, rendering them more appropriate for clinical environments [188].

EMG-based intention detection examines hand muscle activity through the collection of invasive (iEMG) or surface electromyography (sEMG) signals to forecast finger movement intentions in stroke patients. This approach depends on muscular and neural activity, providing high sensitivity, simplicity, and the ability to measure multiple channels. It facilitates accurate finger control. However, it is vulnerable to noise interference and may lead to muscle fatigue. Signals are generally obtained through Myo armbands or sEMG sensors for gesture detection and SRG actuation (Figure 3b) [57,100,104,115,175,189,190,191]. Studies demonstrate that EMG-based intent recognition accurately classifies patient hand postures and movements, improves recognition precision, and achieves device control during the complete hand motion process [192]. Numerous systems employ forearm sEMG monitoring to evaluate muscle activation and deduce patient intent [75], and enabling incorporation into wearable SRGs [193,194], thereby promoting active patient engagement in opening and grasping exercises [64].

EOG-based intention detection monitors eye movements by measuring potential differences in the skin surrounding the eyes, enabling the prediction of hand actions with signal amplitudes between 0.4 and 10 mV. This method is independent of hand nerves or muscles, rendering it appropriate for patients with significantly impaired or absent hand function. EOG provides benefits including straightforward detection, enhanced real-time functionality, ease of wear, and durability. Challenges such as baseline drift, artifact signals, and the difficulty in distinguishing commands frequently occur, resulting in its common integration with other intent detection methods. Integrating EOG with EEG enhances the reliability and safety of continuous grasp control, as EOG signals can be disrupted by unintended movements [195]. Vision-based intent decoding, such as through the use of an eye tracker, facilitates a direct association between intent recognition and target objects [196]. Furthermore, the control of SRG grasping using binocular eye-tracking has been validated without the need for intricate intent recognition algorithms [189].

Voice-based intention detection facilitates the control of SRGs through voice commands, providing a natural interaction experience, ease of use, and the elimination of the need for additional wearable devices. Nonetheless, it exhibits significant vulnerability to environmental noise, slower response times, and necessitates basic language proficiency from stroke patients. Clinical trials have shown that integrating voice-based intent detection with EMG control significantly improves rehabilitation outcomes for post-stroke hand function [197]. Voice-controlled SRG facilitate real-time hand movement training [189]. Voice interaction frameworks utilizing large language models offer patients enhanced intuitive control interfaces, effectively tackling the issue of ADLs intent recognition for stroke patients employing soft gloves [198].

#### 3.1.4. Computer Vision

Methods for intent detection based on computer vision [196,199,200] utilize depth cameras or video cameras to capture the hand motions of stroke patients. They recognize hand gestures, critical points, motion trajectories, and hand-object interactions to deduce movement intent and regulate SRGs. This technology is independent of muscles or nerves, embodying a non-invasive, highly adaptable, and promising approach to intent detection.

Visual intent detection enhances intuitiveness and offers greater inclusivity for patients. In comparison to EEG or EMG, it demonstrates less vulnerability to noise interference, thereby improving object recognition precision. Chen et al. [196] propose a vision-guided soft exoskeleton that facilitates autonomous rehabilitation training execution without the need for further calibration. Cordella et al. [199] presented the Gloreha Sinfonia SRG, including bend sensors and cameras (Figure 3e). It correlates the voltage from bend sensors with angles recorded by an electro-optical camera to measure finger bending angles. Furthermore, in a multi-modal intent recognition approach that integrates machine vision and voice recognition, a Kinect depth camera identifies the pixel locations of the hand, exoskeleton, and gripped object in real time, and a Bluetooth headset-microphone device concurrently records patient audio. Voice commands reduce safety hazards linked to errors from single-modal systems (Figure 3f) [200]. Vision-guided SRG-based hand rehabilitation training can reduce various interference effects while ensuring safety, consequently improving gripping accuracy and motion precision to enhance rehabilitation efficiency.

The integration of several intent detection methods substantially improves the precision and utility of soft gloves in stroke hand rehabilitation, including buttons, sensors, vision [112], EEG and EOG [195], voice and EMG [197], sensors and vision [199], voice and vision [200], EEG and EMG [201], EOG, EEG and EMG (Figure 3g) [202], and so on.

Figure 3 shows the patient intention detection methods of SRGs.

### 3.2. Patient Intent Detection and Control of FES

The integration of intent recognition and FES is commonly utilized to improve hand self-care capabilities in stroke patients [203]. Same as SRGs, primary approaches for intent detection in FES encompass the use of buttons, biological signals, and computer vision.

#### 3.2.1. Button

Button [29,140,147,204] serves as the most straightforward approach for intent detection and control. In the sFES system developed by Popovic et al. [147], therapists initiate hand movements, including reaching or grasping, through button presses. Non-invasive systems make it easier to train finger grasping by combining standard periodic FES with button control [204]. The stimulator is designed to respond to specific button commands, providing stimulation sequences through therapist-activated switches to induce functional hand movements and muscle contractions [29]. Usman et al. [140] utilized a graphical user interface (GUI) featuring clickable buttons to control the initiation, cessation, and mode of electrical stimulation for hand function training in patients.

#### 3.2.2. Sensors

In FES systems, IMU and flexion sensors are frequently employed to identify hand movement intentions in stroke patients.

**Figure 4:**
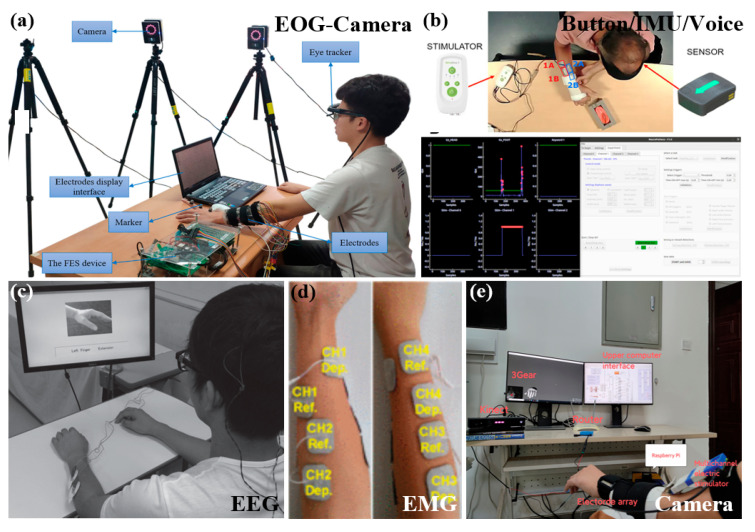
Patient intention detection methods of FES. (**a**) EOG/Camera-based intention detection of FES [150]. Copyright 2021, Taylor & Francie. (**b**) Button/IMU/Voice-based intention detection and control of FES [206]. Reproduced with permission from Le Guillou et al., BioMedical Engineering OnLine, Published by Springer Nature, 2024. (**c**) EEG-based intention detection of FES [215]. Reproduced with permission from Mukaino et al., Journal of Rehabilitation Medicine (JRM), Published by Foundation for Rehabilitation Information, 2014. (**d**) EMG-based intention detection and control of FES [219]. Reproduced with permission from Bi et al., IEEE Access, Published by IEEE, 2020. (**e**) Camera-based intention detection of FES [228]. Reproduced with permission from Zhao et al., the Intelligent Robotics and Applications: 15th International Conference, ICIRA, Published by Springer Nature, 2022.

IMU precisely captures authentic hand movement intentions. Their lightweight, reliable, and cost-effective characteristics have resulted in their use in various FES rehabilitation studies [205,206]. Parnandi et al. [207] utilized 11 IMUs to gather movement data from the upper limbs, including hands, of stroke patients during object manipulation. Le Guillou et al. [206] utilized NeuroPrehens software to identify patients’ movement intentions through input signals from EMG, IMU, switch, and microphone sensors, which activated surface FES (Figure 4b). O’Dwyer et al. [208] and Usman et al. [140] integrated flexion sensors with FES to assess finger bending for the purpose of predicting movement intentions. Tacca et al. [209] integrated bend sensors with high-density EMG fusion to estimate hand and finger joint angles and predict continuous hand positions during movement. Overall, sensor-based monitoring of finger joint movements serves as a method for translating patient intent into the control of rehabilitation devices.

To improve intent recognition accuracy in the presence of electrical noise and weak biological signals, Cao et al. [203] integrated a wearable musculoskeletal ultrasound system with FES, which facilitated real-time, high-precision decoding of hand intent and clinically enhanced rehabilitation outcomes for patients’ hand function. This alignment with patient intent enables FES systems to effectively identify and respond to movement intentions, and minimize muscle fatigue.

#### 3.2.3. Biological Signals

Wessberg et al. [210] demonstrated the feasibility of predicting hand trajectories in real-time through the use of biological signals. Biological signal-based intent detection methods encompass EEG, EMG, EOG, and voice analysis.

In FES, EEG is predominantly utilized via a BCI. BCI-FES has been extensively utilized in rehabilitation by directly decoding motor intentions from stroke patients while simultaneously activating FES. This enhances rehabilitation efficiency by offering more inclusive training for individuals with severe hand impairments [211,212,213,214,215]. Chen et al. [213] created an EEG-based intent recognition BCI-FES system aimed at improving hand function following a stroke. BCI-FES based on motor imagery and neurofeedback has also been implemented in hand training for chronic stroke patients (Figure 4c) [214,215], and facilitates neural remodeling [216].

EMG serves as a direct biological signal indicative of a patient’s intent for muscle movement, improving the sensitivity of FES triggers. Nonetheless, it applies solely to patients who maintain residual muscle activation [40]. EMG-FES systems interpret hand intentions by recording and analyzing electrical signals produced during muscle contraction [209,217,218,219]. Toledo-Peral et al. [218] employed sEMG to forecast grip force and activate FES. Zeng et al. [217] utilized sEMG for the recognition of hand movement intentions, achieving closed-loop FES control in upper limb rehabilitation. Bi et al. [219] utilized EMG signals as stimuli for FES to facilitate rehabilitation training in patients (Figure 4d). The FITFES device, as proposed by Crepaldi et al. [205], integrates EMG and IMU to capture finger movements, achieving muscle-controlled FES.

Methods for intention detection based on EOG [150,220] or voice [206,221,222] provide benefits including significant resistance to interference and real-time functionality, which can improve the robustness of FES systems. Research indicates the potential of EOG in intent recognition and control within FES systems for stroke patients [220]. Wang et al. [150] employed EOG for intent recognition to control a multi-point surface FES system, thereby reducing hand rehabilitation duration (Figure 4a). Le Guillou et al. [206] demonstrated the feasibility of using voice for patient intent recognition and FES control by evaluating the reliability and usability of nine self-triggering methods in their study. Overall, EOG is frequently utilized for direction selection and confirmation signals, rendering it appropriate as an auxiliary strategy in multi-modal fusion. Voice commands provide benefits in natural interaction and semantic expression, categorizing them as advanced task instructions. Nonetheless, the intricate nature of intent recognition and the challenges associated with long-term stability result in a relatively restricted application of both modalities in functional electrical stimulation rehabilitation for hands affected by stroke.

#### 3.2.4. Computer Vision

FES systems based on computational vision intent detection [223,224,225,226,227,228] identify hand postures and movements via RGB or depth cameras, activating FES to facilitate non-contact recognition of intricate intentions independent of bio-signals, thus improving training safety. However, stroke patients frequently demonstrate impaired hand movements, and variables including lighting changes, hand occlusion, and system latency diminish the precision of visual intent recognition.

Simonsen et al. [223] and Zhou et al. [228] utilized Kinect to monitor and capture patients’ finger movement intentions for FES control, achieving hand opening and grasping tasks (Figure 4e). Alimanova et al. [224] and Santamaría-Vázquez et al. [225] employed Leap Motion for gesture detection, incorporating it with virtual reality to improve the efficiency of hand rehabilitation and the progress of recovery via therapeutic games. Wang et al. [150] utilized cameras to record hand motion data, which functioned as both calibration and enhancement for EOG. Bhagat and Ruppa [227] developed a computer vision-based FES system for home rehabilitation. This system utilizes cameras to capture patients’ hand movement intentions in real time, enabling the control of FES to assist fingers in grasping different objects. Lin et al. [226] introduced a closed-loop FES system that incorporates visual interaction perception, featuring a visual perception module for sliding detection and intent recognition. Detection of 21 critical hand position points enhances patients’ autonomous grasping abilities and improves overall hand function.

Figure 4 shows the patient intention detection methods of FES.

### 3.3. Patient Intent Detection and Control of HHRSs

Given the limited research available on HHRSs for stroke patients, related intention detection techniques are likewise relatively underexplored, rendering this area of considerable research importance. Intention detection techniques of HHRSs can predominantly be classified into sensor-based and bio-signal-based methodologies.

#### 3.3.1. Sensors

Soft wearable devices with flexible sensors can pick up on small changes in the angles of finger joints. This makes them suitable for controlling HHRSs that use soft robotics and FES. Hartopanu et al. [158] employed bending sensors to measure finger flexion angles as proxies for patients’ intended hand movements, relaying data to a control unit for the assessment of stroke patients’ rehabilitation progress. Neto et al. [159] developed a portable hybrid system that combines tendon-driven soft gloves with FES. They used optical force myography (FMG) sensors to figure out what patients wanted to perform with their hands. The hybrid FES soft glove system evaluated by Popescu et al. [229] also used bend sensors to provide feedback on finger movement intention, which helped them finish hand rehabilitation exercises.

#### 3.3.2. Biological Signals

The use of biological signals as an intent detection method in a HHRS necessitates compatibility with two rehabilitation modalities. Two of the most popular signals are EEG [230] and EMG [157,159,162,231,232].

**Figure 5:**
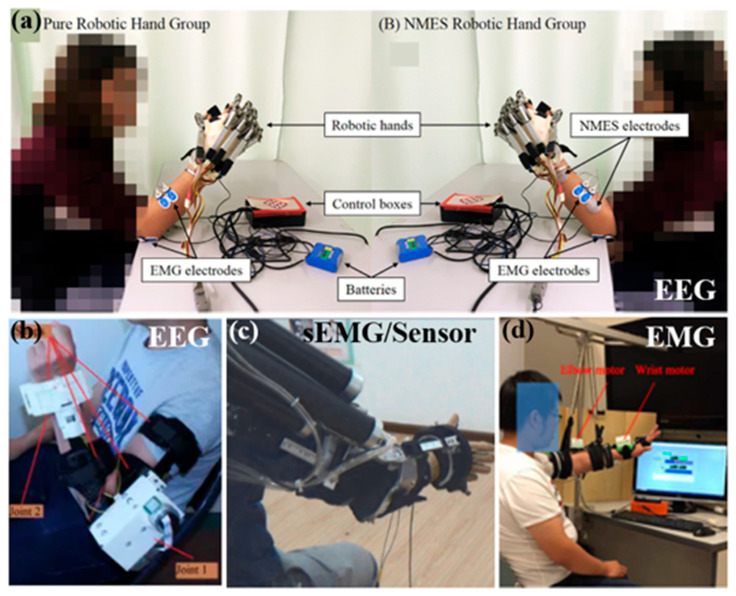
Patient intention detection methods of HHRSs. (**a**) EMG-based intention detection and control of FES-robotic systems [157]. Reproduced with permission from Hu et al., Biomedical Signal Processing and Control, Published by Elsevier, 2020. (**b**) EEG-based intention detection and control of upper-limb robotic-FES system [230]. Reproduced with permission from Elnady et al., Frontiers in Human Neuroscience, Published by Frontiers Media S.A., 2015. (**c**) sEMG/Sensor-based intention detection of a SRG powered by pneumatic artificial muscles and an FES array system [231]. Reproduced with permission from Tu et al., Journal of Healthcare Engineering, Published by Wiley, 2017. (**d**) EMG-based intention detection and control of neuromuscular electrical stimulation and robot hybrid system [233]. Reproduced with permission from Rong et al., Journal of NeuroEngineering and Rehabilitation, Published by Springer Nature, 2017.

To aid in the rehabilitation of stroke patients using a upper-limb robotic-FES system, Elnady et al. [230] developed the BCI-FES system, which uses EEG produced by motor imagery for intent recognition (Figure 5b). The subjects’ experimental results showed BCI-FES/robotic systems for hand function rehabilitation are feasible. A SRG powered by pneumatic artificial muscles and an FES array system was developed by Tu et al. [231]. Coordinated control of hand extension and grasping training is made possible by identifying patients’ active hand movement intentions through sEMG, position sensor, and force sensors (Figure 5c). It is similar to the work of Rong et al. (Figure 5d) [233]. When it occurred to chronic stroke rehabilitation, an EMG-driven NMES robotic hand system performed better than pure robotic systems, according to a clinical comparison trial conducted by Huang et al. (Figure 5a) [157]. Another study [232] has also demonstrated that sEMG-driven FES-robotic systems have positive effects on upper limb rehabilitation training.

Figure 5 shows the patient intention detection methods of HHRSs.

## 4. Control Algorithms

Upon identifying patient intent, the hand rehabilitation system can utilize it as control commands to operate rehabilitation devices directly. For example, it can establish EMG thresholds to differentiate between opening and closing, activate EOG commands through blinking, or employ computer vision to assess hand-object distance. Alternatively, it may enhance algorithms before controlling devices for training completion.

The control algorithm is tasked with the real-time and accurate translation of intent signals into the force and posture of SRGs or FES. Rehabilitation robots need to achieve a balance between providing effective assistance and reducing the likelihood of errors. Therefore, control algorithms must exhibit safety, real-time performance, stability, resistance to interference, and high robustness. Current control methods primarily include traditional algorithms and artificial intelligence (AI) algorithms, with AI emphasizing deep learning (DL) and machine learning (ML). Traditional control strategies differ based on the particular rehabilitation device employed. Table 3, Table 4 and Table 5, respectively, present the main technical characteristics of the devices used or developed in each study within the SRGs, FES, and HHRSs categories.

### 4.1. Control Algorithms of SRGs

Considering the nonlinear properties of SRGs and the intrinsic variability of patient intent, the development of effective control algorithms is essential for enhancing hand function in stroke patients.

#### 4.1.1. Traditional Algorithms

Compared to AI algorithms, traditional control algorithms do not necessitate extensive training data and demonstrate enhanced stability, controllability, and generalization performance. However, they are inadequate for the processing of sophisticated, high-dimensional data.

Yap et al. [72,99] analyzed pneumatic sensor data based on proportional-integral-derivative (PID) control to facilitate SRG in assisting patients with ADLs requiring grasping. Given the straightforward nature of PD control implementation, Ivanescu et al. [234] utilized conventional PD control for flexible SRG and examined their biomechanics. Rakhtala et al. [95] employed a model-based PID controller for the closed-loop regulation of input pressure and finger flexion angle in SRGs. Ismail et al. [57] employed proportional-integral compensation for digit flexion and extension control. Chen et al. [197] uses voice as a switch to control the device status, and controls the soft robotic glove by combining EMG thresholds with a proportional control algorithm. Overall, PID-based approaches are adaptable and straightforward to implement, but do not possess the ability to adapt to human intent.

To improve control performance, Polygerinos et al. [23] implemented sliding mode control (SMC) to modulate hydraulic pressure, although they did not consider system uncertainties. Tang et al. [104] designed a SRG based the probabilistic model-based learning control, facilitating patients’ daily functional hand movements. However, its proficiency in fine motor skills has yet to be validated. Li et al. [86] utilized a model-free anti-disturbance control approach incorporating an extended state observer for SRG, achieving repetitive hand extension training with enhanced disturbance rejection and improved transient control performance relative to PID algorithms.

Researchers have also examined the nonlinearity and uncertainty intrinsic to SRGs. Jeong et al. [51] utilized sEMG as a trigger threshold, implementing isometric, isokinetic, and impedance control strategies to operate the SUN Exo-Glove for hand strength training. Copaci et al. [115] employed k-Nearest Neighbor (KNN) to classify sEMG signals and integrated this approach with bilinear PID (BPID) control of SMA-actuated soft mittens to achieve intent recognition and accurate control.

#### 4.1.2. Artificial Intelligence Algorithms

The remaining hand signals of stroke patients are frequently weak and noisy, necessitating AI algorithms to accurately extract meaningful intentions from sensors, bio-signals, or visual data for the control of SRGs. Deep learning [235,236] and machine learning [60,168,194,237,238] substantially improve control precision and resilience [179]. In contrast to traditional algorithms, AI methodologies are more adept at handling intricate nonlinear interactions, allowing for autonomous feature extraction, exhibiting robust noise resistance, and promoting multi-modal fusion control.

Secciani et al. [238] introduced the Point-in-Polygon algorithm, which, while akin to ML, offers enhanced robustness for the classification of sEMG signals for the control of wearable hand exoskeletons. Chen et al. [58] evaluated various ML techniques for gesture classification and developed a SRG to facilitate precise hand movements. Sierotowicz et al. [194] employed advanced ML algorithms to examine sEMG signals, converting them into finger movements via low-level admittance control. This established closed-loop control for a compliant assistive glove, exhibiting robust performance with both ridge regression and random Fourier characteristics. Lin et al. [168] employed ML to identify fingertip pressure signals for SRG-assisted musical performance, achieving the greatest accuracy with artificial neural network (ANN) classification. Liu et al. [235] utilized Transformer-based decoding of EEG data to control SRG, therefore augmenting neural plasticity. Zhou et al. [236] presented adaptive position control via radial basis function neural networks to enhance the precision of pneumatic SRG-assisted grasping.

Table 3 presents the main technical characteristics of the devices used or developed in each study within the SRG category.

**Table 3:** Technical characteristics of each study within the SRG category.

Actuation Type	Force Transmission	User Intent Detection	Control Strategy	Active Fingers	Weight	Max Force	Reference
Hydraulic	Water	Hydraulic pressure sensor	Feedback Control	All	<3.5 Kg	8 N	[23]
TSA	String	IMU	VR system	All	290 g	17 N	[53]
Motor	Tendon	Button/sEMG	PI	Index Middle Ring	729 g	6 N	[57]
Motor	Cable	sEMG	Neural Network	All	258 g	10 N	[65]
pneumatic	Air	Task-oriented/sEMG	Probabilistic model-based learning control	All	180 g	-	[104]
pneumatic	Air	Magnetic field intensity	Threshold	All	150 g	9.8 N	[105]
SMA	Wire	Bending sensor- Camera/Touchable screen	PI	All	490 g	75 N	[112]
SMA	SMA	EMG	BPID ^(a)^	All	-	17.5 N	[115]
pneumatic	Air	EEG	Threshold	All	-	-	[121]
Motor	Cable	Pressure sensor	P	Thumb Index Middle	≈700 g	20 N	[168]
Motor	Tendon	EOG	-	Thumb Middle Ring	-	-	[189]
Motor	Cable	Voice-EMG	Threshold	All	<400 g	300 N	[197]
Motor	Cable	EEG-EMG	LDA	All	-	-	[201]
Pneumatic	Air	Flexible sensor/Motion capture system	RBFNNO ^(b)^	All	-	-	[236]
Motor	Cable	Pressure sensor	P	Thumb Index Middle	≈700 g	20 N	[23]

^(a)^ BPID: Bilinear Proportional Integral Derivative; ^(b)^ RBFNNO: Radial Basis Function Neural Network Adaptive Control Algorithm.

### 4.2. Control Algorithms of FES

In FES hand rehabilitation, electrical stimulation by itself lacks the capacity for intelligent operation. It necessitates the implementation of control algorithms to ensure safe, natural, and effective rehabilitation results, thereby achieving the intelligent functioning and closed-loop regulation of the FES system.

#### 4.2.1. Traditional Algorithms

Exell et al. [239] integrated an iterative learning control (ILC) algorithm with a gesture recognition program to translate recognized movements into FES modes, thereby improving fine motor skills in the fingers of stroke patients. Hughes et al. [240] utilized a model-based ILC algorithm to leverage upper limb position data obtained from Kinect^®^ for the control of finger extension and grasping in a FES system. Zhou et al. utilized a genetic algorithm-optimized ILC to enhance precision in stimulating multi-joint finger movements within FES systems, demonstrating clinical validation of its efficacy in improving hand function. ILC exhibited superior tracking control performance and efficient regulation for systems characterized by repetitive motion.

Wolf et al. [141,241] integrated feedforward-feedback algorithms to regulate a FES system for reaching movements in patients with upper limb paralysis, illustrating the system’s capability for goal-directed motion. Usman et al. [140] introduced an auto-calibration algorithm utilizing sensory feedback and prior knowledge to autonomously identify optimal stimulation parameters and electrode pad placements through motion feedback sensors, facilitating selective finger control. Guo et al. [242] employed sEMG as an input for intent, implementing closed-loop grasping for FES via predictive control and convolutional optimization, establishing a foundation for new intelligent electrical stimulation models. Lin et al. [226] improved patients’ autonomous grasping and fine manipulation using feedback control and adaptive closed-loop FES systems, thereby improving the ADLs in home settings. Cao et al. [203] utilized FFT to process ultrasound intention signals in the context of FES-assisted hand rehabilitation.

#### 4.2.2. Artificial Intelligence Algorithms

Xu et al. [243] employed a compact convolutional neural network based on transfer learning for BCI-FES in the detection of rehabilitation intentions in stroke patients and the generation of control signals, demonstrating improved robustness. Bhagat et al. [227] used a convolutional neural network-based object detection to control FES, achieving grasping support for various objects. Parnandi et al. [207] compared four machine learning algorithms and determined that Linear Discriminant Analysis (LDA) exhibited the highest classification accuracy and practicality. Bi et al. [219] utilized LDA for the classification of sEMG gestures and mapped them with stimulation channels. Validated through six stroke patients, the system demonstrated effective execution of actions such as grasping.

Table 4 presents the main technical characteristics of the devices used or developed in each study within the FES category.

**Table 4:** Technical characteristics of each study within the FES category.

Actuation Type	User Intent Detection	Control Strategy	Function	Channel	Current [mA]	Frequency [Hz]	Reference
imFES	Button/goniometer/touch pad	Button/Touch pad	Reach, Grasp, and Release	8	5–40	-	[132]
imFES	EMG	Threshold	ADLs	4	-	-	[138]
sFES	Flex sensor	Auto-calibration	Grasping/Hand closing	32	5–15	25	[140]
sFES	Button	-	Grasping/Hand extension	3	8–50	20–40	[147]
sFES	EOG-Camera	GUI	ADLs	25	-	-	[150]
sFES	Ultrasound	Threshold	Motor function reconstruction	4	10–21	30	[203]
sFES	EMG/IMU/Vosion	-	Grasping	2	-	-	[206]
sFES	EEG	Threshold	Hand extension	2	10–25	16–30	[211]
sFES	EEG	NFB ^(a)^	ADLs	-	2	20	[214]
sFES	sEMG	-	Grip strength	2	0–15	50	[217]
sFES	sEMG	LDA	Grasping/Hand opening/Precision grasp	8	-	-	[218]
sFES	sEMG	LDA	Grasping/Finger extension	4	<30	20–60	[219]
sFES	Camera	Deep learning	Grasping	4	-	-	[227]
sFES	Camera	Closed-loop feedback	Grasping	4	-	30	[226]

^(a)^ NFB: Neurofeedback.

### 4.3. Control Algorithms of HHRSs

To date, hybrid hand rehabilitation devices have undergone only limited experimental evaluation, and some systems have not been tested as fully integrated platforms, such as tactile robotic gloves [158,244] and ALEx [245]. In many studies, exoskeletons were tested only on the designers themselves or on a small number of healthy subjects. Overall, most reported hybrid systems have not been evaluated on more than 10 participants, nor have they undergone more than 20 training sessions [36]. A limited number of studies have conducted more systematic clinical evaluations. For example, the FES/Robot Hand system [233] was tested on 11 chronic stroke patients who completed 20 training sessions, with clinical outcomes assessed using measures such as the Modified Ashworth Scale and the Fugl–Meyer Assessment. Similarly, the NMES-based soft hand exoskeleton developed by Nam et al. [162] was evaluated in 15 stroke patients after 20 training sessions, demonstrating that hybrid systems can effectively support self-directed upper-limb rehabilitation in stroke patients.

Overall, most hybrid hand rehabilitation systems have been validated primarily through functional feasibility testing, and no commercially available hybrid hand rehabilitation devices currently exist. The high cost and system complexity of hybrid rehabilitation systems remain major barriers to large-scale clinical evaluation and clinical translation.

The limited availability of relevant research presents challenges in fully validating control algorithms for HHRSs in both experimental and clinical contexts. Existing control methods for upper limb exoskeleton-FES hybrid systems, along with the characteristics of SRGs, provide a basis for inferring feasible control strategies. The appropriate algorithms need be chosen according to the rehabilitation requirements of stroke patients.

Hartopanu et al. [158] utilized a button proportional control method to modulate the bending positions of fingers, thereby facilitating various finger movements and accelerating the rehabilitation process. Neto et al. [159] introduced a hybrid control strategy that activates FES while preserving grip with a SRG, thereby minimizing muscle fatigue and facilitating home-based rehabilitation. Agnanto et al. [160] utilized a multi-input multi-output fuzzy logic controller to regulate the SRG-FES hybrid system, and demonstrated its viability through preliminary experiments conducted with five healthy subjects, suggesting potential applicability for hemiplegic or muscle-weakened stroke patients. Takenaka et al. [246] utilized a probabilistic neural network to analyze EMG signals as indicators of patient intent, successfully achieving hybrid control between robotics and FES, which was validated in experiments with 12 healthy participants.

Tu et al. [231] employed an ILC algorithm to regulate a hybrid system that integrates an upper-limb exoskeleton powered by pneumatic artificial muscles with FES. The exoskeleton facilitated arm movement, whereas FES controlled finger grasping. Three subjects demonstrated their capacity to collaboratively engage in active reaching and grasping training. Dalla Gasperina et al. [247] developed a collaborative control scheme to allocate the necessary torque between an elbow robot and FES. The motor control component incorporates weight-compensated feedforward and feedback impedance controllers, whereas the FES control component employs trial-by-trial ILC. This approach achieves accurate trajectory tracking and confirms the successful integration of FES with robotic actuation.

**Figure 6:**
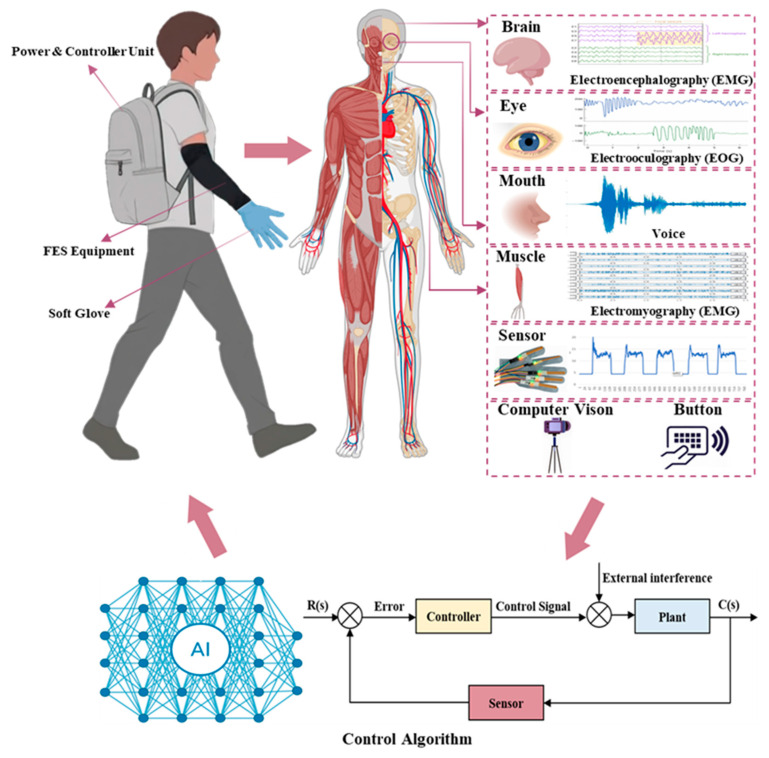
Schematic diagram of the hybrid hand rehabilitation system.

The overall workflow of the hybrid hand rehabilitation system is shown in Figure 6.

Table 5 presents the main technical characteristics of the devices used or developed in each study within the HHRS category.

Model-based controllers remain suitable for low-dimensional, repetitive rehabilitation tasks, whereas learning-based methods are more advantageous for intention decoding and multi-modal fusion under high uncertainty.

**Table 5:** Technical characteristics of each study within the HHRS category.

Actuator	FES	Control Strategy	User Intent Detection	Function	Weight	Advantage	Disadvantage	Portability	Reference
Cable-SRG	Motion stim 8	Button proportional	Button-Flex sensor	Finger bending motion	56 gactuator	balanced control between FES and exoskeleton	Slow response time	No	[158]
Cable-SRG	STIMSHIELD	Hybrid control	Optical FMG sensor	Grasp	-	Delaying muscle fatigue	Cannot control 5 fingers individually	Yes	[159]
Motor-SRG	2 channels	MIMO-FLC ^(a)^	Flex sensor	Grasp	-	Delaying muscle fatigue	Cannot control 5 fingers individually	No	[160]
Breg T-Scope Elbow brace	Reha Stim I	Proportional control of predefined trajectories	EEG	Stretching and grasping of hand	1 Kg	Wear quickly within 30 s	Predefined trajectory	Yes	[230]
RUPERT	Reha Stim2	ILC	EMG	active reach-to-grasp trainings	-	active reach-to-grasp trainings	Large volume, high complexity, and high cost	-	[231]
Motor Upper Robot (no hand)	4 channels	Adaptivecontrol	sEMG	Elbow and wrist flexion/extension, hand opening	895 g	Improve the muscular coordination at the elbow, wrist and fingers	No assistance from the system for finger flexion	No	[233]
Elbow Robot	Rehamove3	Impedance control-ILC	Torque sensor	Repetitive flexion of elbow joint	-	Easy to transfer to clinical	-	No	[247]

^(a)^ MIMO-FLC: Multi-Input–Multi-Output Fuzzy Logic Controller.

## 5. Discussion

SRGs and FES have shown potential in improving hand rehabilitation efficiency. However, further optimization is necessary to develop lightweight, wearable, and safe portable rehabilitation solutions that align with patients’ daily living and work requirements [37]. Restoration of hand motor function and reorganization of motor neurons are primary goals in post-stroke hand rehabilitation. HHRSs that integrate SRGs with FES utilize the benefits of compliant mechanical support and neuromuscular activation, facilitating synergistic interactions between external movement guidance and internal neural reconstruction. Hybrid hand rehabilitation has emerged as a significant area of research [248]. Although the number of HHRSs is still limited, this integration represents a qualitatively different rehabilitation paradigm, rather than a simple combination of two technologies.

This review discusses the current state and uses of SRGs, FES, and HHRSs. It focuses on actuation methods, intention detection and control approaches, and control algorithms. Additionally, we present four key issues and recommendations.

### 5.1. The Portability of Hybrid Hand Rehabilitation Systems

SRGs serve as an exceptional wearable basis for hybrid hand rehabilitation systems owing to their flexibility, lightweight construction, and superior comfort. As a non-mechanical neuromuscular drive technique, FES provides benefits like significant portability and superior energy economy. Nonetheless, the conventional driving topologies of SRGs and FES discussed in this paper continue to encounter limitations. Soft robotic actuators often rely on external motors, air pumps, control modules, or tube assemblies, resulting in cumbersome and heavy systems that are difficult to carry and wear. FES activation necessitates customized electrode positioning for specific arm muscles, with stimulation intensity influenced by electrode placement and resistance fluctuations. Ongoing electrical stimulation also causes muscular exhaustion, restricting training length. These factors cumulatively undermine system portability, rendering current HHRSs [162,231,246] often cumbersome, stationary, and substantial weight. Although certain devices [158] fulfill size specifications, they demonstrate relatively slow response speeds. HHRSs significantly diminish superfluous muscle activity, mitigate fatigue accumulation, and surpass single SRGs or FES systems in hand load capacity, movement precision, and grasp reliability [231], thereby illustrating distinct functional complementarity and superior rehabilitation performance.

Portability can be quantitatively assessed by system weight, volume, power consumption, and operational autonomy. For wearable hand devices, a commonly accepted portability guideline is that the on-hand component should not exceed 500 g [249], and any external power supply or control units should not exceed 3 kg [23]. Hybrid systems typically require multiple external modules and high energy supply, leading to increased weight and bulkiness and limited battery life, which reduces their suitability for home-based or daily use. Therefore, compared with standalone SRGs or FES systems, the portability of current HHRSs is generally poor.

Moreover, the substantial energy consumption of HHRSs impedes their portability. SRGs demonstrate significant energy requirements, irrespective of the use of electric, pneumatic, or hydraulic actuators. The persistent electrical stimulation output from FES devices elevates battery demand, leading to both increased system weight and restricted operational duration. This affects stroke victims’ daily activities and ongoing rehabilitation training.

Despite considerable problems in portability, HHRSs have distinct advantages over single rehabilitation devices in terms of naturalness, efficacy, and functional compensation. They provide a more promising wearable rehabilitation alternative for patients with post-stroke hand movement impairments.

### 5.2. Multi-Modal Intention Detection Approaches

Intent detection serves as a crucial element in rehabilitation systems for stroke patients’ hands, as it directly influences the ability of rehabilitation devices to swiftly and accurately interpret a patient’s movement intent, thereby activating appropriate SSRG assistance or FES. Stroke patients exhibit significant variability in the extent of hand nerve damage, often resulting in unstable intention-related signals and insufficient hand motor function. These challenges render single-modality intention detection methods inadequate for fulfilling clinical rehabilitation requirements. Consequently, it is essential to combine the advantages of various detection methods to improve the accuracy of identifying patients’ hand intentions and to enhance system reliability. For example, computer vision-based hand rehabilitation systems predominantly exist at the technology validation stage (TRL 5 or 6), with limited progression to system development (TRL 7 or 8) and an even smaller number achieving full system testing and deployment (TRL 9) [250].

This review demonstrates that single-modality intent detection continues to be the primary method in stroke hand rehabilitation, while multi-modal approaches are increasingly being integrated into devices like SRGs [102,150,195,197,199,200,201]. Multi-modal intention detection is largely lacking in FES and HHRSs. This is mainly due to the inherent high nonlinearity and considerable inter-individual variability of FES systems. Misinterpretation of multi-modal signals can result in inconsistent stimulation intensity or movement control, thereby affecting rehabilitation outcomes and introducing safety risks.

Multi-modal intention detection should not be treated as equal-weight fusion, but rather as a hierarchical or supervisory structure, especially when FES is involved. Multi-modal intention detection provides a thorough understanding of movement intention in stroke hand rehabilitation. However, it presents challenges, including multi-source heterogeneity, signal synchronization, noise robustness, real-time processing, and model complexity. The variations in temporal scales, sampling rates, and noise characteristics among signals hinder cross-modal fusion and feature alignment.

Nevertheless, multi-modal approaches in post-stroke hand rehabilitation can enhance the accuracy and robustness of intent recognition, mitigate the risk of false triggering in FES or SRGs control, and facilitate safer, more natural, and personalized assisted training. The integration of HHRSs with multi-modal intention detection to construct a closed-loop intelligent rehabilitation architecture is a significant future direction for stroke rehabilitation. Thus, the development of understandable, safe, and individually adaptive multi-modal intent detection and control methods, while ensuring system robustness and real-time performance, continues to be a fundamental challenge in contemporary research.

### 5.3. Multiple Rehabilitation Exercise Modes and the Ability to Switch Freely of HHRSs

Hand dysfunction in stroke patients primarily presents as spasticity or paralysis, with rehabilitation efforts concentrating on finger extension and grasping. HHRSs delay muscle atrophy, provide grasping assistance, support various ADLs, and promote neural plasticity through active movement, facilitating more comprehensive recovery of hand function.

From a system perspective, patient intent signals are used to enable flexible switching among rehabilitation modes. When mechanical assistance is required, intention signals coordinate the SRG and FES; when mechanical support is unnecessary, FES alone delivers electrical stimulation to the target muscles to execute the desired movements; conversely, in grasp-oriented tasks that require only robotic assistance, intention signals can directly control the SRG. In practical applications, the primary rehabilitation technology should be selected according to the patient’s recovery stage and functional needs, while the intensity of FES or the auxiliary level of the SRG can be adjusted to optimize the overall efficiency of the HHRS. Furthermore, when intent recognition or task execution is hindered by noise interference, the system utilizes control algorithms to suppress errors and achieve closed-loop compensation, thereby enhancing system stability and integration.

Overall, HHRSs enhance control precision and motion repeatability, supporting more refined finger movements. They can address complex rehabilitation requirements, cover training across different recovery stages, and enhance movement naturalness, system robustness, and patient comfort, highlighting considerable clinical potential. Nevertheless, challenges persist in intent detection accuracy, control complexity, and safety, influenced by factors including multi-actuator collaboration, mode-switching stability, reliability of multi-modal intent recognition, and variability in patient states. Consequently, the attainment of stable, safe, and seamless multi-modal switching is essential for the progression of HHRSs towards clinical application and home-based rehabilitation.

### 5.4. Home-Based Rehabilitation

Stroke patients can enhance muscle strength and facilitate the reconstruction of hand neural plasticity through active engagement in rehabilitation training. While certain SRGs have been commercialized, these devices frequently exhibit limited functionality due to the strict requirements for portability and ease of use in home settings, which are generally inadequate for assisting patients with complex ADLs. In contrast, FES is a proven technique for neurological rehabilitation. The gadget is small and more portable, and its stimulation level may be readily adjusted. Rehabilitation programs can be tailored to patients’ needs in order to assist them in regaining hand motor function. However, the use of FES in home-based hand rehabilitation is still quite restricted.

HHRSs exhibit significant benefits for hand function recovery. However, research in this area is still in the preliminary stages of development. Current systems encounter issues including intricate actuator drive mechanisms, variable intent detection methods, and challenges in coordinating control strategies. Furthermore, factors such as bulky size, significant weight, and limited mobility compromise their overall structure, making them inappropriate for home-based rehabilitation in the short term. To achieve effective home-based hand rehabilitation that addresses patients’ daily living activity requirements, future design and development of hybrid rehabilitation systems must prioritize lightweight construction, portability, and user-friendly operation.

Based on the above considerations, future hybrid hand rehabilitation systems for home-based stroke recovery should prioritize lightweight actuation, compact power modules, and simplified user interfaces. A feasible system concept is illustrated in Figure 6, where a pneumatic soft glove provides compliant assistance while minimizing weight and avoiding secondary injury, and high-density surface FES is applied to the forearm to activate targeted muscles. The hand-worn burden can be reduced by integrating a micro air pump, compact pressure regulator, control unit, and battery into a wearable backpack, with a centralized short flexible air tubing. Soft actuators should adopt low-pressure pneumatic operation combined with efficient valves (response time < 20 ms) to reduce energy consumption and extend battery life.

To support independent home use, the system should be powered by high energy-density lithium-ion batteries, targeting at least 2–3 h of operation per session. Energy management strategies such as sleep modes, adaptive stimulation intensity, and demand-based actuation can further prolong runtime, while real-time monitoring of data with automatic shutdown ensures safety. User-friendly interaction can be achieved through a smartphone app for mode selection, progress tracking, and personalized training, supplemented by voice commands or single-button control for patients with limited dexterity. Automatic mode switching based on intention detection (e.g., IMU or pressure sensors, and optionally EMG when it does not interfere with FES) can improve training compliance. Finally, a modular quick-don/doff architecture comprising a SRG module, forearm sFES band, and backpack containing control and power units can reduce setup time and facilitate customization and maintenance.

## 6. Conclusions

Hand dysfunction represents a prevalent and enduring functional deficit post-stroke, significantly affecting patients’ independence in daily activities and their mental health. Hand rehabilitation enhances muscle strength and motor coordination while promoting neural plasticity, which increases the probability of long-term functional recovery in the hand. Thus, the advancement of portable, efficient, and wearable rehabilitation technologies that promote active patient engagement is crucial for the rehabilitation of hand impairments following a stroke.

This review systematically analyzes the relevant SRGs, FES, and HHRSs from three perspectives of drive mechanisms, patient intention detection methods, and control strategies. Research demonstrates that SRGs provide benefits, including compliance, safety, high comfort, and durability, whereas FES directly stimulates muscles and nerves, facilitating active rehabilitation. The combination of these two approaches creates a complementary hybrid drive mechanism, showing potential and feasibility in improving rehabilitation training efficacy, promoting neural plasticity, and enhancing hand motor function. HHRSs are a significant future direction for rehabilitation technology, necessitating further detailed investigation.

HHRSs are currently in the early stages of development and encounter challenges including bulkiness, complex actuation, inadequate reliability in multi-modal intent detection, and difficulties in unifying control strategies. Hybrid rehabilitation methods, in contrast to single-system approaches, can address a wider spectrum of patient hand functional levels, facilitate diverse movement patterns, and present potential benefits in enhancing active participation, closed-loop control, and personalized rehabilitation. Advancements in soft materials, flexible electronics, low-power actuation, wireless communication, multi-modal fusion sensing, and AI control algorithms suggest that HHRSs may enable effective wearable, home-based, and intelligent rehabilitation in the future. This will enhance the clinical application and translational significance of recovery in hand function following a stroke.

## Figures and Tables

**Table 1:** Related study reviews from the last 8 years.

Group	Reference	Intended Population	Affected Function	Main Conclusion
SRG	[14]	Stroke and SCI	Hand	Hand exoskeletons have limitations in terms of DOF, sensing ability, and control algorithms
	[20]	Stroke	Hand	SRG can improve the functional ability of upper limbs in stroke patients
	[37]	Stroke	Hand	Design choices meeting each requirement were identified, but quantitative analysis could not be performed
	[38]	Stroke and SCI	Hand	Soft gloves have great potential but require further advances in actuation–sensing–control integration.
FES	[27]	Stroke and SCI	Upper-limb Hand	BCI-controlled FES can be more effective than FES alone in rehabilitation
	[39]	Stroke and SCI	Hand	Transcutaneous multi-channel FES of the upper extremity is safe in SCI and stroke patients
	[40]	stroke	Shoulder Elbow Wrist Hand	Verified the feasibility and effectiveness of an FES-based upper limb stroke rehabilitation system
	[41]	Stroke and SCI	Lower-limb Upper-limb Hand	FES therapy can effectively restore motor function in patients with stroke and SCI and holds promising potential
HHRS	[26]	SCI	Hand	Soft robotics and FES wearable devices are promising for hand function recovery, yet face some limitations
	[35]	Stroke	Hand	Robotic gloves and FES are more effective than traditional therapies in improving specific tasks in post-stroke patients
	[36]	Stroke	Shoulder Elbow Hand	Hybrid exoskeletons are benefit for post-stroke hand rehabilitation but are still in early development

**Table 2:** Advantages and disadvantages of four separate actuation mechanisms of SRGs.

Actuation Type	Definition	Advantages	Disadvantages	Response	Durability	Actuators
Motor	Convert electrical energy into mechanical energy, providing rotational or linear motion to drive the deformation of flexible structures	(1) Precise control capabilities (2) Suitable for complex motion (3) Simple structure (4) Pollution-free (5) Rapid signal response (6) High load capacity	(1) The motor’s inherent rigidity limits the deformation capability of flexible structures (2) Heavyweight (3) Bulky size (4) High inertia	Quickly	High	Tendon, String, Cable, Spring
Hydraulic	Driving the deformation or motion of soft structures by controlling fluid pressure and flow	(1) High power output and wide-range motion control (2) suitable for heavy-duty applications	(1) Requiring sealing and leak-proof design (2) Liquid supply and discharge necessitate consideration of environmental and safety concerns (3) Bulky size (4) High noise levels	Quickly	High	Rubber, Spring, Artificial muscle, Fabric, Silicone
Pneumatic	Using pressurized air to cause finger’s actuators flexion or extension	(1) Stepless speed regulation (2) Wide range of DOF and range of motion (ROM) (3) Lightweight (4) High safety (5) Portable (6) Low cost (7) Simple structure	(1) Requires precise pressure and flow control systems (2) Difficult to control accurately (3) Suitable for low-power drives	Medium	Medium	Fabric, silicone Rubber, Pneumatic artificial muscle, etc.
SMA	By controlling the temperature of the SMA, its shape can be altered and maintained	(1) Simple drive and control systems (2) safety and comfort (3) Rapid response (4) High-efficiency shape transformation	(1) High cost (2) Complex manufacturing and high integration (3) Accurate temperature control	Slowly	Low	-

## Data Availability

The data that support the findings of this study are available from the authors upon reasonable request.
